# Factors Associated With Restraint Application in Children and Adolescents With Intellectual and Developmental Disabilities Displaying Severe Challenging Behavior

**DOI:** 10.1177/01454455261434863

**Published:** 2026-04-15

**Authors:** Asude Sumeyye Ayvaci, Alison Dorothea Cox, Daniel Mitteer

**Affiliations:** 1 Brock University, St. Catharines, ON, Canada; 2 Rutgers University Center for Autism Research, Somerset, NJ, USA

**Keywords:** emergency physical restraint, intellectual and developmental disability, restraint determinants, severe challenging behavior

## Abstract

Emergency physical restraints may still be widely used, with prevalence rates ranging from 11% to 78% across different service sectors (Fitton & Jones, 2020). At times, behavior analysts may recommend restraint when severe challenging behavior poses significant safety risks (e.g., intense aggression causing severe tissue damage; Foxx & Meindl, 2007; Vollmer et al., 2011). Most existing physical restraint literature featuring child and adolescent participants focuses on variables that behavior-analytic interventions do not manipulate (e.g., sex, diagnosis). Additionally, this literature most often features inpatient clinical populations with psychiatric conditions as opposed to those with intellectual and developmental disabilities. The current study applied a multi-level analysis informed by retrospective outpatient data (*N* = 12) from children and adolescents with intellectual and developmental disability who required emergency physical restraints. The study aimed to (a) examine and report on participant and restraint application characteristics and trends, and (b) determine if challenging behavior severity at intake predicted latency to restraint-application. According to the descriptive analysis, most participants were experiencing polypharmacy (i.e., prescribed at least three concurrent psychotropic medications), had been assigned moderate to high scores on challenging behavior severity, and primarily exhibited behavior maintained by access to tangibles or multiple reinforcers. Regarding restraint characteristics, the average restraint rate was 9.11 per 100 service hours, with most participants described as calm during restraint application. Typically, more than two staff members applied the restraints. Regression results indicated that challenging behavior severity scores significantly predicted latency to the first restraint application. Clinical implications related to these outcomes are discussed.

## Introduction

Demographic research suggests that up to 50% of individuals with intellectual and developmental disabilities engage in challenging behavior, including aggression, property destruction, or self-injurious behavior (SIB) such as head hitting, self-mutilation, or pica (e.g., Kurtz et al., 2020; Newcomb & Hagopian, 2018). A smaller portion, approximately 5% to 10%, exhibit severe challenging behavior, such as high-risk aggression or pica (e.g., consuming razor blades, batteries, magnets), that may necessitate restrictive interventions to prevent significant harm (e.g., deep lacerations, near-fatal incidents, broken limbs; Ekinci et al., 2021; Foxx & Meindl, 2007; Kurtz et al., 2020; Thillainathan et al., 2024). These severe forms of challenging behavior can endanger the health and safety of both the individual and caregivers and can also restrict access to community involvement and daily activities (Emerson & Einfeld, 2011).

Treatment for severe challenging behavior typically involves psychopharmacological treatments, behavior-analytic interventions, and/or a combination of the two (Cox & Virues-Ortega, 2022). Psychotropic medications (i.e., chemical restraints) are substances that cross the blood–brain barrier and impact neuronal activity, which can, in turn, influence behavior, mood, or thought processes (Dalla et al., 2022). When psychotropic medications are prescribed to manage challenging behavior, individuals can be prescribed more than one psychotropic medication concurrently, which is referred to as polypharmacy (Masnoon et al., 2017). Polypharmacy is frequently observed in this population and has been associated with an increased risk of experiencing significant adverse side effects (e.g., seizures, agitation; Charlot et al., 2020; Sawyer et al., 2014). Due to these risks, psychotropic medications may not be suitable for all individuals. Further, when a comorbid psychiatric diagnosis is absent, research demonstrating psychotropic medication efficacy is underdeveloped, and clinically administration may be considered controversial (e.g., Acton, 2022; Deb et al., 2023).

Behavior-analytic interventions often enact *function-based* treatments so that learners may be encouraged to engage in appropriate alternatives that serve the same purpose as the challenging behavior (e.g., Oliver & Richards, 2015; Pierce & Cheney, 2017). Behavior-analytic approaches such as differential reinforcement, functional communication training, and noncontingent reinforcement are some examples of common strategies used to treat challenging behavior (see Cooper et al., 2020; Pierce & Cheney, 2017). Punishment procedures, like response blocking (e.g., Mitteer et al., 2015) and response interruption and redirection (e.g., Saini et al., 2015), may also be used concurrently with reinforcement-based approaches. However, when reinforcement and decelerative strategies are ineffective or pose safety concerns, restrictive interventions, such as physical restraint and mechanical restraint, may be necessary to prevent harm while supporting the development of adaptive skills (Cox et al., 2025; Luiselli, 2012; Thillainathan et al., 2024). Restraints, including physical restraint, mechanical restraint, and seclusion, are defined as interventions that limit movement to prevent harm (Jones et al., 2007; Vollmer et al., 2011). In Ontario, the Patient Restraint Minimization Act (PRMA; Legislative Assembly of Ontario, 2001) states that restraints may be used to prevent serious injury following the failure of other treatments. The *Professional and Ethical Compliance Code* for Behavior Analysts Certification Board [BACB] (2020) and Vollmer et al. (2011) also provide recommendations around its use and corresponding fading.

### Physical Restraint Prevalence and Characteristics

Physical restraint may be categorized into two types: therapeutic and emergency restraint (Luiselli, 2012). The former may be part of a behavior support plan and used strategically with clear criteria for application and release. The purpose of their application is to reduce challenging behavior and thus make it possible to initiate teaching adaptive skills (Thillainathan et al., 2024). Once stabilized, implementers are expected to execute a plan to fade or eliminate their use (Cox et al., 2025; Foxx & Meindl, 2007). Although demographic research on therapeutic restraint is limited, some studies have described positive outcomes around their application and corresponding fading (e.g., Cox et al., 2025; Langone et al., 2014; Thillainathan et al., 2024).

By contrast, emergency restraints are applied temporarily to manage severe challenging behavior, particularly when there is an immediate risk of injury. These restraints are typically released once implementers determine that the danger has passed (Luiselli, 2012). Recent demographic works indicated significant variation in the prevalence of emergency restraint use across different settings, with rates ranging from 11% to 78% across various service sectors (Fitton & Jones, 2020). Moreover, restraint durations varied widely, from as short as 2 min to as long as 1,458 min over a 3-month period (Fitton & Jones, 2020). Despite ongoing efforts to limit the use of emergency restraints, they appear to remain a common approach.

### Restraint-Application Considerations

Importantly, restraint application can carry significant risks, including asphyxiation and deep vein thrombosis (Funayama & Takata, 2020). The application of vague or subjective criteria (e.g., releasing a restraint when the client appears to demonstrate calm affect without objective measures of calm) for terminating restraints can exacerbate these risks. Conversely, premature release may necessitate reapplication, increasing the likelihood of injury to implementers as well as the client (Jones et al., 2007). The risk of injury during application must be balanced with the risks associated with permitting the challenging behavior episode to continue uninhibited. These risks can be mitigated by implementing objective release criteria. One example is behavior-contingent release, where support personnel are instructed to remove restraints based on the client meeting specific behavioral benchmarks. These benchmarks may include responding to simple questions or completing designated sequences or activities. Another release criterion is fixed-time release, where support personnel may initiate restraint removal after a set duration, regardless of client behavior (Luiselli, 2012).

While physical restraint may be necessary to maintain safety in certain situations, the goal should be to minimize and/or eliminate its use (Vollmer et al., 2011). Research aiming to refine behavior-analytic interventions to expedite treatment gains may serve to facilitate reduced reliance on physical restraint (e.g., Petursson & Eldevik, 2019). Another way to work toward minimizing reliance on restraint application may be identifying client risk factors (i.e., restraint determinants) associated with their application. Establishing this literature base could mean that, for individuals who present with risk predictors, behavior analysts could make strategic intervention and resource (i.e., personnel) decisions early on that may serve to minimize or possibly circumvent their application.

Predictive modeling of challenging behavior and treatment outcomes are becoming more commonplace among behavior-analytic research. For examples, Edgemon and Rapp (2024) demonstrated how conditional probability analyses can identify temporal patterns (e.g., days of the week and times of day) that may be associated with increased incidences of challenging behavior in juvenile justice settings. Work like this may afford proactive resource allocation coinciding with periods associated with a higher likelihood of challenging behavior. Similarly, Joslyn and Morris (2023) highlighted the utility of risk ratios as a practical quantitative method to estimate behavior-environment relations, which can improve the precision of behavioral predictions in both research and practice. Moreover, Falligant and Hagopian (2020) integrated precision medicine concepts into behavior analysis by identifying predictive behavioral markers that may forecast individual response to functional communication training, thereby facilitating individualized treatment planning. Extending this line of work, Morris and Joslyn (2025) applied precision medicine techniques to predict trajectories of challenging behavior (i.e., aggression). Their work offered a framework for allocating clinical resources more effectively based on behavioral markers. Collectively, recent prediction model research exemplifies a growing trend in behavior analysis toward using data-driven models to anticipate challenging behavior and optimize intervention strategies, which holds promise for enhancing both preventive efforts and treatment efficacy. Collectively, prediction research shows promise as it relates to improved client well-being.

### Existing Restraint Determinant Literature

Existing research on restraint determinants involving children and adolescents conducted in Finland found that younger age, male sex, aggression, conduct disorder, attention-deficit/hyperactivity disorder (ADHD), and autism spectrum disorder correlated with restraint application in psychiatric inpatient children (Sourander et al., 2002). Similarly, studies in Australia (e.g., Crocker et al., 2010) and Canada (e.g., Stewart et al., 2010) have identified that the diagnosis of disruptive behavior disorders, high scores on risk assessments, psychotropic medication use, younger age, and self-injury increase the likelihood of restraint. In the United States, Tompsett et al. (2011) linked previous hospitalization and a history of aggression with restraint applications. In Australia, Duke et al. (2014) identified predictors such as physical aggression, early restraint use, and developmental disabilities. More recently, in Norway, Furre et al. (2017) associated low psychosocial functioning, prolonged hospital admissions, and psychotropic drug prescriptions with restraint episodes in psychiatric inpatient children.

At first glance, the demographic research collection on this topic may suggest a comprehensive examination of this phenomenon. However, some glaring limitations may exist. First, the research involving children and adolescents primarily features participants with psychiatric diagnoses in *psychiatric* settings—a clinical population that should be considered distinct from individuals with intellectual disabilities or autism spectrum disorders (e.g., Furre et al., 2017). Second, existing research on predictors of restraint for individuals with intellectual and developmental disabilities primarily features adults aged 27 to 47 years old (e.g., Lundström et al., 2011; Scheirs et al., 2012). This may limit generalizability to children and adolescents, where developmental and physical differences may markedly impact restraint application and associated predictors. Moreover, while existing research has reported participant challenging behavior severity, it was infrequently assessed at intake (e.g., Crocker et al., 2010). Thus, the potential role of challenging behavior severity as a restraint predictor remains elusive. Further, psychotropic medication status, including the presence of polypharmacy, was underreported despite its known relevance to restraint risk (Charlot et al., 2020). Finally, existing studies generally focus on examining the topography of challenging behavior (e.g., aggression) as a predictor without considering its function.

Regarding methodological limitations, one of the most important gaps may be the inconsistent definitions and measurement methods for restraint application across studies, which likely hinders comparisons (Pértega & Holmberg, 2023). Relatedly, many studies have failed to operationally define what constituted a single physical restraint episode (e.g., reapplications as separate episodes versus considering them part of the same restraint event). This can impact construct validity and consistency in data interpretation (Slife et al., 2016). Another limitation may be that some studies have combined multiple restraint types (e.g., physical, chemical) into a single restrictive intervention category (e.g., Duke et al., 2014). This may reduce precision in risk-assessment research. Finally, studies relying on secondary informant data often failed to control for treatment duration and other confounding factors, such as attendance variability due to funding or scheduling limitations. For example, some studies involving adults have mainly relied on indirect, questionnaire-based data collected from staff or family through proxy reporting (e.g., Lundström et al., 2011). This approach is vulnerable to bias (e.g., subjectivity, absence of contextual information, potential for false positives) and may poorly align with direct-observation measures (Fryling & Baires, 2016). While rate-based measures might control some confounds, they are still susceptible to biases introduced by time-based variables (e.g., history effects, maturation; Ledford & Gast, 2018). Using latency as a dependent variable, which measures the time from appointment onset to the first restraint application, could offer a more sensitive metric across participants (Cooper et al., 2020).

The current study attempted to address the research gaps listed above as we examined patterns among select participant characteristics and restraint application factors. Specifically, we aimed to (1) examine the characteristics of children and adolescents with intellectual and developmental disabilities who required emergency physical restraint, (2) describe typical features of emergency physical restraint applications within the study sample, and (3) assess whether challenging behavior severity at intake was associated with latency to the first emergency physical restraint application.

## Method

Ethics clearance was obtained from the research ethics boards of both participating institutions. Caregivers with adolescents receiving services from a hospital-affiliated intensive-outpatient clinic in the Northeastern United States provided consent for their child’s clinical data to be collected and included in a research database (File: Pro2019002168). Brock University research ethics board granted clearance for researchers to access de-identified records from this database (file: 22-076).

### Participants and Setting

The research team reviewed 2 years of client records from the intensive-outpatient clinic. The study included data from participants who met the following criteria (a) 19 years old or younger, (b) experienced an emergency physical restraint application while receiving services, and c) had a diagnosis of intellectual and developmental disability. Researchers identified 12 children and adolescents who met these criteria and analyzed their clinical records. To maintain confidentiality, pseudonyms were assigned: Ben, Bobby, Clark, Freddy, Gerald, Glady, Ken, Leonard, Marcus, Marshall, Michael, and Tim. These participants received outpatient behavioral services between September 2022 and April 2024.

The intensive outpatient clinic operated 5 days a week. Participants attended for either 3 or 6 hr daily. Appointments took place in 3 m × 3 m therapy rooms with padded walls and floors, one-way observation mirrors, and two-way intercom systems. Each appointment was staffed by at least two registered behavior technicians. Clinical oversight was available daily, as the cases were supervised by one Board Certified Behavior Analyst (BCBA) and one Board Certified Behavior Analyst-Doctoral (BCBA-D). Individualized approaches were implemented for each participant to minimize restraint application as much as possible (e.g., blocking, protective equipment). Staff responsible for restraint application received training through an extensive in-house restraint program and were only permitted to implement restraints following mastery during competency assessments.

### Materials

At intake, a BCBA-D administered the Destructive Behavior Severity Scale (DBSS; Fisher et al., 2013, Fisher et al., 2022) to the participant’s legal guardian to assess challenging behavior severity and help inform (a) program eligibility and (b) dose and duration of services. DBSS is comprised of six domains: aggression, property destruction, SIB, disruptive behavior, pica, and injury to self or others. That is, this scale measures dangerous or destructive behaviors that pose risks to individuals (e.g., pica, SIB), others (e.g., aggression), or the environment (e.g., property destruction) and is suitable for assessing various topographies of challenging behavior. Although the DBSS has not yet been validated, clinicians may use it to (a) assess challenging behavior severity in individuals with intellectual and/or developmental disabilities, and (b) determine the need for protective equipment. See Fisher et al. (2022) for the publicly available scale as part of their Severe Behavior Program Toolkit.

At a minimum, staff wore padded arm guards with child-sized shin guards underneath and chest protectors while carrying a small or large pad (depending on the size of the participant) for aggression or SIB. For any participant who displayed head-directed aggression, staff wore padded helmets with face shields and relevant hair protection (e.g., swim caps) to minimize the risk of hair pulls. When biting was a referral concern, staff wore cut-proof gloves underneath their arm guards. Please see Figure 7.2 and Table 1 in Irwin Helvey et al. (2024) for images and examples of these protective-equipment items. During his admission, Ben voluntarily wore a padded helmet provided by his family to minimize the risk of face-directed SIB.

**Table 1.** Destructive Behavior Severity Scale Outcomes Across Participants.

Category	Level 1 (%)	Level 2 (%)	Level 3 (%)	Level 4 (%)	NA (%)
Injury Risk	0	8.33	8.33	33.34	50
Aggression	8.33	8.33	25	50	8.33
Property Destruction	0	16.67	8.33	75	0
SIB	0	25	25	41.67	8.33
Pica	8.33	25	8.33	8.33	50
Disruption	8.33	25	0	25	41.67

*Note.* This table shows results for participant characteristic variables coded using a single response coding. NA = not applicable, indicating the participant did not exhibit this challenging-behavior topography.

### Restraints

Per clinic policy and relevant regulations, the clinical team had to attempt less-intrusive strategies (e.g., response blocking, protective equipment, modifying the environment, verbal cues) prior to restraint and to document these strategies in the electronic medical record. In every case, clinical teams attempted to implement these strategies (e.g., removing furniture, using additional blocking pads, warning the client that staff may have to help hold them to keep them calm) before implementing a restraint. Staff requested supervisor presence when restraint became likely, at which point at least one BCBA-D and a relevant medical provider (e.g., advanced practice nurse; APN) would provide support (e.g., offer additional non-restraint strategies) and make the call to order and initiate restraint. Emergency procedures and restraint criteria remained in place regardless of whether the client was in a formal assessment or treatment session or during free time.

Restraints were implemented based on precise criteria developed by clinicians (e.g., two instances of head-directed SIB within 10 s). Whenever possible, clinicians collaborated with caregivers and participants to inform application and release criteria. Generally, clinicians initiated restraints following challenging behavior that posed a danger to the client or staff based on its severity (e.g., head-directed aggression that posed risk of concussion, eye-targeted SIB) or a local rate (e.g., five instances of headbanging within 30 s) and terminated restraints following 30 s or 60 s without co-occurring challenging behavior (e.g., attempts to headbang or bite others during the restraint).

Most restraints involved holding the participant’s arms at the forearms or biceps to minimize SIB or aggression, and often the legs at the thighs or shins to minimize aggression in the form of kicking. Restraints occurred in standing, seated, or laying positions depending on the posture of the client at the time of the restraint initiation (e.g., standing if the client was standing) with these positions changing according to safety needs (e.g., seated in a chair if the client dropped their weight while standing in a restraint). No restraints involved a prone position. For participants with headbanging or biting, restraints occasionally involved a therapist holding their hands slightly above the participant’s forehead to limit mobility upward.

At least one BCBA-D remained present during the entirety of the restraint, with the medical provider documenting the restraint and overseeing the patient’s physical and emotional needs (e.g., assessing vitals). No medical attention was necessary during or following any restraint. Clinicians informed caregivers of each restraint use and attempted to debrief participants on restraint use when possible.

### Data Collection

After obtaining de-identified clinical records, we coded relevant clinical data across two main categories. The first category was *participant characteristics*, which included variables such as DBSS at intake, age, polypharmacy status, behavior-assessment outcomes (i.e., challenging behavior function), and diagnosis (see Participant Characteristics below for more details). The second category was *restraint characteristics*. We extracted data from participant *restraint notes* to code these variables. These notes were clinical records (i.e., patient charts) completed by an APN at the time the restraint was applied. Restraint notes contained pertinent information regarding restraint applications, including but not limited to patient intervention response, restraint procedure start and end times, and any instances of restraint *reapplication* (i.e., more than one instance of restraint application recorded on a restraint note). See Restraint Characteristics below for fulsome details. As per clinic policy, once the restraint note is completed, the information is immediately uploaded to a secure database. All physical restraint applications were considered emergency procedures; and for concision, they will henceforth be referred to as *restraints*.

### Data Extraction and Coding

The data-extraction process involved coding (a) participant-characteristic data, and (b) restraint-characteristic data from restraint-application notes. We used a categorical coding approach for some variables (e.g., restraint follow-up mood, challenging behavior function) and continuous coding approach for others (e.g., age, inter-response interval of restraint application). Following methods outlined in Ayvaci et al. (2024), we leveraged both coding schemes to enhance data-extraction precision. For the categorical variables, we applied either single-response or multiple-response coding where appropriate. For the single-response coding, we assigned numeric values to each individual category. For example, if Ben’s medication regime at intake included four psychotropic medications, this variable would be assigned a value of 3, denoting polypharmacy presence.

For categories with multiple responses (i.e., subcategories), each subcategory was either assigned a value of 1 to indicate its presence or left blank to indicate its absence. For example, Ken exhibited both SIB and aggression. Therefore, each topography (i.e., subcategory) was marked with a 1 to denote its presence within the challenging behavior topography category.

For multi-response coding items, we applied a *percentage-based approach* because total percentages in such coding schemes can exceed 100% when multiple responses are allowed. This means that, when computing percentage bases, the numerator was the total count of occurrences of a given subcategory (e.g., SIB or aggression), and the denominator was the total number of coded responses across the overarching category (e.g., challenging behavior topography) rather than the number of participants, multiplied by 100. For participants wherein coding multiple subcategories within a category were required, we counted each selection independently. For instance, if 12 participants have multiple challenging behavior topographies (e.g., aggression, SIB, pica), with 8 demonstrating SIB, 7 demonstrating aggression, and 5 demonstrating pica, then percentages would be 80%, 70%, and 50%, respectively, totaling 200%. It follows that, in calculating multi-response outcomes, the denominator is the sum of occurrence in each subcategory (interested readers may refer to Ayvaci et al., 2024; McBeath, 2020, for details). Following data extraction and coding, we imported variables informing the regression analysis (i.e., challenging behavior severity [i.e., predictor variable] and latency [i.e., outcome variable]) into the Statistical Package for the Social Sciences (SPSS).

#### Participant Characteristics

The following variables were coded as per single-response coding.

##### Polypharmacy Presence

Data were coded as (a) only one medication, (b) two medications, (c) polypharmacy, and (d) no medication.

##### Functional Analysis

We coded whether a functional analysis (FA) was conducted. This information was derived from the participant’s medical record in which BCBA-D clinicians described the relevant assessments conducted with a patient and their outcomes (i.e., identified functions). FAs involved at least one test and control condition using a single-case experimental design. In all cases analyzed for this study, FAs included multiple test and control conditions within multielement or reversal designs. Clinician discretion determined the number of conditions and other procedural details (e.g., use of latency measures, providing consequences for a single or multiple topographies).

##### Challenging Behavior Severity at Intake

The DBSS includes four severity levels. Each domain is scored separately on a scale from 1 (least severe) to 4 (most severe). The severity score associated with each domain was coded as follows: (a) Level 1, (b) Level 2, (c) Level 3, and (d) Level 4.

##### Challenging Behavior Severity Median at Intake

To generate one coefficient per participant, we considered the advantages and disadvantages of average versus median (central tendencies) and decided to calculate a median score informed by each DBSS category (e.g., aggression severity, pica severity). Interested readers may review Field (2024) for introductory content on central tendencies, or Khorana et al. (2023) for further detail on central tendency selection. This variable served as the independent variable for the regression analysis.

The following variables were coded as multi-response.

##### Challenging Behavior Topography

Challenging behavior topography, as originally labeled by the clinicians at the intensive outpatient clinic, was categorized in accordance with Cox et al. (2021). This variable was comprised of nine categories, including: (a) aggression, (b) SIB, (c) property destruction, (d) elopement, (e) disruption, (f) pica, (g) inappropriate sexual behavior, and (h) suicidal ideation.

##### Challenging Behavior Function

Eight categories delineated challenging behavior function, including: (a) inconclusive (e.g., Hagopian et al., 2007), (b) automatic, (c) escape, (d) tangible, (e) attention, (f) multiply controlled (indicating that the behavior was maintained by more than one function), (g) undifferentiated (i.e., the FA results were undifferentiated, e.g., Cox & Friedel, 2022), and (h) specialized (e.g., mand compliance). Raw FA results were not shared with Brock University researchers. Instead, clinicians’ (e.g., BCBA-D) visual interpretations from the partnering intensive outpatient clinic were used to inform this variable, which was often based on expert visual inspection and structured criteria.

##### Diagnosis

Participants’ diagnoses, according to their medical records, were categorized into the following: (a) autism spectrum disorder, (b) obsessive-compulsive disorder, (c) Tourette’s, (d) ADHD, (e) post-traumatic stress disorder, (f) generalized anxiety disorder, (g) postural tachycardia syndrome, (h) chromosomal disorder, (i) seizure disorder, and (j) other (e.g., mood disorder).

The following variables were coded as continuous variables.

##### Age

Participants’ chronological age in years at intake.

##### Total Service Duration

The date of the first appointment marked appointment start, and the date of the last appointment marked service termination. In calculating this variable, we excluded holidays, missed appointments, weekends etc. Of the 12 participants in the study, 10 met their clinical goals and graduated from the program. That is, they no longer required outpatient services. These goals were set by clinicians, legal guardians, and, when possible, the clients themselves. For Ben and Ken, their families chose to move them to residential placements when these rare placement opportunities presented themselves during their intensive-outpatient clinical admissions. Generally, residential-care beds are limited and often ensure continued intensive support.

#### Restraint-Application Characteristics

The following variables were coded as per single-response coding.

##### Number of Staff Involved

The number of staff required to implement a restraint application was coded as: (a) one to two staff involved and (b) more than two staff involved.

The following variables were coded as multi-response.

##### Response to Intervention

For this variable, restraint-note coders (i.e., APNs) conducted face-to-face evaluations immediately upon and throughout restraint application. Participants’ emotional responses and their interactions with their surroundings and other individuals during this time were observed and recorded. At times, coders documented more than one concurrent reaction, which included: (a) calm, (b) cooperative, (c) paradoxical, (d) uncooperative, (e) hostile, (f) other, and (g) not recorded.

##### Follow-Up Mood

The emotional state documented post-emergency restraint. The emotional state that could be coded was created by multidisciplinary teams at the partnering agency’s intensive-outpatient clinic (e.g., psychiatrists, psychologists). Restraint**-**note coders would conduct face-to-face evaluations several minutes after terminating restraint application during a wellness check, which had to be completed within 60 min according to hospital regulations. Possible participant emotional state (i.e., affect) categories included: (a) euthymic, (b) angry, (c) anxious, (d) irritable, (e) depressed, (f) labile, (g) afraid, (h) euphoric, (i) other, and (j) not recorded. At times, restraint**-**note coders recorded more than one mood concurrently.

The following variables were coded as continuous variables.

##### Total Frequency of Restraint Notes

The presence of a completed restraint note was considered one restraint note. We calculated the total frequency of restraint notes by tallying the number of completed restraint notes while in service.

##### Total Frequency of Restraints

A restraint application (i.e., restraint episode) was considered any instance when the APN documented the restraint procedure start time. This coefficient included restraint reapplications (i.e., re-initiating a restraint after releasing due to participant re-escalation) that may occur *within* a single restraint note. For instance, Ben’s staff implemented a restraint at 09:49 am. The restraint was terminated shortly after, but the team had to reapply a restraint at 09:59 am and again at 10:08 am. In terms of coding, we recorded three instances of restraint application. Once all restraint notes were coded, all instances of restraint application were tallied, which produced a total count coefficient per participant.

##### Total Frequency of Restraint Reapplication

This variable was the total frequency of restraint reapplication per restraint note. That is, each instance of a restraint application that occurred after the initial restraint was counted. For example, in one of Ben’s restraint notes, staff terminated the first restraint at 10:00 am. Following this, the team reapplied a restraint at 10:30 am, and again at 10:40 am. In this situation, two applications within a single restraint note were added to the final tally. Once all restraint notes were coded, all instances of reapplication were tallied. This produced a total restraint reapplication count per participant.

##### Overall Restraint Rate

We calculated this variable by dividing the total frequency of restraints by the total duration of service (in hours) and then multiplying the result by 100 to obtain the rate. For example, Freddy had 31 restraint applications in total across 625.80 hr of service for an overall restraint rate of 5 per 100 service hours.

##### Latency

Latency to first restraint application was the time from the start of services to the first recorded restraint note in hours. This variable served as the dependent variable for the regression analysis.

##### Inter-Response Interval Time of Restraint Applications

This variable was coded as follows. First, latency was excluded from this variable. Thus, the time from the participant’s appointment start time (i.e., the start of their service) to the beginning of their first restraint procedure (i.e., latency) was not included in the averages and ranges calculated. The remainder of the inter-response interval times were computed by calculating the time between each restraint procedure end time and the next restraint procedure start time. The following is a hypothetical example for clarity. A participant began services on Tuesday, June 18th, and generally attended 6 hr appointments from 9:30 am to 3:30 pm daily. If this participant required a restraint for the first time on Tuesday, June 18th at 2:48 pm, the latency between the start of service and the first restraint was 5.3 hr (i.e., 5 hr 18 min). If this restraint procedure ended at 2:54 pm on the same day, to produce the first inter-response interval, we used this time (2:54 pm) until the next restraint was recorded. That is, if this participant required another restraint at 3:13 pm on the same day, then the time between the end of the first restraint (i.e., 2:54 pm) and the start of the next one (3:13 pm) was calculated. This resulted in an inter-response time of 0.32 hr (or 19 min). This calculation considered participants’ appointment hours. Calculations were adjusted to account for appointment start delays (e.g., participant late arriving to appointment) and/or absences (e.g., holidays, illness, weekends).

### Interobserver Agreement

#### Participant and Restraint Variables

To calculate interobserver agreement (IOA) for data-extraction variables, we used Google Randomizer to select which participant data would be coded by a second independent rater (33.33% of participants; *N* = 4). The independent raters were two graduate-level research assistants who were trained to code study variables via a modified behavioral skills training approach (see Parsons et al., 2013). To calculate IOA, we used an item-by-item method. This involved dividing the total number of agreed-upon coded items by the total number of items rated, then multiplying by 100 (Cooper et al., 2020). Rater agreement was defined as both raters assigning the same value to the same variable. The IOA for participant-characteristic variables was 92.33% (range 80%–100%). For restraint-note variables, it was 97.25% (range 87.50%–100%). Most discrepancies were attributed to varying levels of Excel proficiency among the research assistants which led to differences in calculations between them and the first author. In short, we did not observe any reliable error patterns.

#### Regression Analysis Variables

The independent rater for regression analysis variables was the project supervisor, and second author. She extracted and coded 100% of the variables that were exported to SPSS to execute a regression analysis (see below for details). We used exact count-per-item wherein each data entry per participant was treated as an item (Cooper et al., 2020). We computed IOA by dividing the total number of exact agreements by the total number of participants and multiplying by 100. Agreement was defined as both raters producing the same value for each participant. The agreement between raters was 100%.

## Data Analysis

We performed descriptive statistics to summarize participant characteristics (i.e., question one) and restraint characteristics (i.e., Question two), as well as a simple linear regression to determine whether challenging behavior severity (i.e., independent variable) was associated with latency to first restraint application (i.e., dependent variable; Question three).

### Descriptive Analysis

We calculated the descriptive results for the single-response category by summing the frequency of the variable’s occurrence. Descriptive results for multi-response coding items were obtained by calculating the percentage base for each subcategory (e.g., SIB, aggression, property destruction) under a single overarching category (e.g., challenging behavior topography). Finally, to enhance results’ clarity and concision, we leveraged select central tendencies across continuous and categorical variables, where appropriate. For example, Michael had a restraint rate of 0.10, Leonard 0.30, Bobby 0.60, Tim 0.60, Clark 1.80, Glady 2.20, Marcus 4.30, Marshall 6.50, Freddy 5.00, Ken 10.70, Gerald 37.60, and Ben 39.70 per 100 service hours. Thus, the average restraint rate across all participants was 9.11 per 100 service hours (see the Results section for full details). By contrast, we reported the median for variables with large ranges (e.g., Total Frequency of Restraints; see Results), thus affording more robust reporting.

### Regression Analysis & Corresponding Rationale

Regression analysis is a statistical tool used to uncover the relationship between one (i.e., simple regression) or many (i.e., multiple regression) independent variables and the dependent variable (see Field, 2024 for a gentle introduction). For the current project, regression models facilitated quantifying how changes in the predictor variable (i.e., challenging behavior severity) corresponded to changes in the outcome variable (i.e., latency to first restraint). Recommendations in the context of regression analysis suggest at least 10 participants per independent variable (Field, 2024). Thus, the current dataset permitted examining the relationship between one independent variable (challenging behavior severity) and the dependent variable (latency to first restraint).

When conducting a simple linear regression to determine whether a variable predicts another variable (e.g., challenging behavior severity predicting the latency to restraint application), it is important to consider the dataset’s composition (Palmer & O’Connell, 2009). For instance, in the current context, including individuals who did not experience restraint application would not contribute a viable latency value. Thus, including these non-exemplars would introduce a zero-inflation issue, which would distort model estimates (e.g., Campbell, 2021). Analyses that include both participants who experienced restraint application and those who did not typically require alternative statistical approaches, such as logistic regression for event occurrence or survival analysis for time-to-event outcomes (Bewick et al., 2004). Our featured research question required us to narrow the analysis exclusively to exemplars (i.e., those who experienced restraint application).

## Results

### Supplementary Material Statement

In the interest of transparency, corresponding Supplementary Materials offer a detailed summary of select participant and restraint characteristics.

### Descriptive Analysis

#### Participant Characteristics

##### Polypharmacy Presence

Overall, 83.33% of study participants were taking more than three psychotropic medications concurrently. Approximately 16.67% had no known medication status.

##### Functional Analysis

Across the sample, 91.67% had experienced an FA. The team had elected to forego conducting this assessment for Glady (8.33%) due to relevant referral concerns.

##### Challenging Behavior Severity at Intake

Table 1 outlines detailed results of challenging behavior severity at intake. Fifty percent of participants exhibited the highest level of aggression (Level 4), and 75% showed the highest level of property destruction (Level 4). SIB was also prominent, with 41.67% of participants scoring Level 4. In contrast, pica and disruption were coded as less severe.

##### Challenging Behavior Topography

Table 2 provides itemized results for percentage base calculations across participants. The most frequent behavior reported across the sample was aggression (41%). This was followed by property destruction (24%), then SIB (21%).

**Table 2.** Percentage Base Across Participant Challenging Behavior Topography.

Categories	Percentage base (%)	Frequency
Challenging Behavior Topography		
Aggression	41	12
Property Destruction	24	7
SIB	21	6
Elopement	3	1
Disruption	7	2
Pica	0	0
ISB	0	0
Suicidal ideation	3	1

*Note.* This table presents the percentage-based calculations for participant challenging behavior topography, coded using a multiple-response approach. ISB = inappropriate sexual behavior; SIB = self-injurious behavior.

##### Challenging Behavior Function

Access to tangible was observed most frequently, appearing across 29% of the sample. This was followed by multiply controlled functions (24%). Escape from instructions and access to attention was observed 16% of the time, with specialized functions (e.g., mand denial) reported across 8% of the sample. Undifferentiated challenging behavior function was identified 3% of the time, and only 1% had not experienced an FA (see Supplemental Material).

##### Diagnosis

In addition to being diagnosed with an intellectual and developmental disability, the most often observed diagnosis across participants was autism spectrum disorder at 34%. This was followed by an ADHD diagnosis (17%), and seizure disorder (11%). Less frequently observed diagnoses included “other” diagnoses, such as mood disorders (9%). Tourette’s syndrome and chromosomal disorders were reported 6% of client files. Diagnoses of generalized anxiety disorder, post-traumatic stress disorder, major depression, and postural tachycardia syndrome were recorded for 3%, respectively.

Table 3 outlines the results for participant characteristics for continuous variables.

**Table 3.** Select Participant Characteristics.

Participants	Age	Diagnosis	Total service duration (hr)
Ben	14	ASD	362 67
Bobby	17	ASD, ADHD, seizure disorder	723.88
Clark	17	ASD, tic disorder	765.43
Freddy	17	ASD, GAD, seizure disorder	625.80
Gerald	19	ASD	244.55
Glady	15	ASD, ADHD, PTSD, major depression, POTS	136.75
Ken	14	ASD, ADHD, OCD, Tourette’s syndrome	317.62
Leonard	8	ASD, ADHD, ODD, Tourette’s syndrome	359.41
Marcus	14	ASD, ADHD, OCD	599.33
Marshall	13	ASD, DMDS	541.15
Michael	19	ASD, seizure disorder, chromosome 15q11–q13 duplication syndrome	1,580.90
Tim	16	ASD, GAD, ADHD	352.33

*Note.* This table presents the exact age, diagnosis, and total service duration for each participant. ASD = autism spectrum disorder; ADHD = attention deficit and hyperactivity disorder; DMDS = disruptive mood dysregulation disorder; GAD = generalized anxiety disorder; OCD = obsessive-compulsive disorder; POTS = postural tachycardia syndrome.

##### Age

Participant ages ranged from 8.00 to 19.00 years (*M* = 15.25 years).

##### Total Service Duration

The median service duration was 451.91 hr. Service duration ranged from 136.75 hr (i.e., Glady) to 1,580.90 hr (i.e., Michael).

#### Restraint-Application Characteristics

##### Number of Staff Involved

For Gerald, Leonard, Tim, Freddy, and Marcus, more than two staff members were required to implement restraint application 95% of the time or more. Restraint applications for Ken and Marshall required more than two staff members more than 80% of the time. For Bobby and Clark, more than two staff members were involved in 75% of restraint applications. Glady’s notes suggested 50% of restraint applications involved one to two staff members, and 50% of restraint applications required more than two staff members (see Supplemental Materials for detailed results on the number of support staff involved in restraint notes per participant).

##### Response to Intervention

Michael’s and Tim’s responses to the intervention (i.e., restraint) were calm and cooperative 100% of the time. Ben, Bobby, Clark, Freddy, Gerald, Ken, Marcus, and Marshall predominantly displayed calm and cooperative responses 86.5% of the time on average. For Glady, the team observed paradoxical, uncooperative, and hostile responses, each occurring 33% of the time, while Leonard exhibited uncooperative responses to the restraint 100% of the time. Refer to Table 4 for fulsome results.

**Table 4.** Percentage Base Across Select Coded Variables.

Part.	Coded variable	Response options	% Base	Freq.	Coded variable	Response options	% Base	Freq.
Ben	Response to Intervention	Calm	52	80	Follow-up Mood	Euthymic	24	29
		Cooperative	32	50		Angry	15	18
		Paradoxical	3	4		Anxious	27	33
		Uncooperative	10	16		Irritable	13	16
		Hostile	1	1		Depressed	15	18
		Not recorded	3	4		Labile	0	0
						Afraid	2	2
						Euphoric	2	2
						Other	0	0
						Not recorded	3	4
Bobby	Response to Intervention	Calm	33	2	Follow-up Mood	Euthymic	0	0
		Cooperative	33	2		Angry	17	1
		Paradoxical	0	0		Anxious	33	2
		Uncooperative	33	2		Irritable	33	2
		Hostile	0	0		Depressed	0	0
		Other	0	0		Labile	17	1
		Not recorded	0	0		Afraid	0	0
						Euphoric	0	0
						Other	0	0
						Not recorded	0	0
Clark	Response to Intervention	Calm	33	6	Follow-up Mood	Euthymic	0	0
		Cooperative	44	8		Angry	20	4
		Paradoxical	11	2		Anxious	30	6
		Uncooperative	0	0		Irritable	30	6
		Hostile	6	1		Depressed	0	0
		Other	0	0		Labile	10	2
		Not recorded	6	1		Afraid	5	1
						Euphoric	0	0
						Other	0	0
						Not recorded	5	1
Freddy	Response to Intervention	Calm	48	21	Follow-up Mood	Euthymic	3	1
		Cooperative	48	21		Angry	3	1
		Paradoxical	0	0		Anxious	29	11
		Uncooperative	0	0		Irritable	34	13
		Hostile	2	1		Depressed	8	3
		Other	0	0		Labile	16	6
		Not recorded	2	1		Afraid	0	0
						Euphoric	0	0
						Other	0	0
						Not recorded	8	3
Gerald	Response to Intervention	Calm	0	0	Follow-up Mood	Euthymic	0	0
		Cooperative	0	0		Angry	14	15
		Paradoxical	33	1		Anxious	3	3
		Uncooperative	33	1		Irritable	24	28
		Hostile	33	1		Depressed	0	0
		Other	0	0		Labile	54	60
		Not recorded	0	0		Afraid	0	0
						Euphoric	0	0
						Other	5	5
						Not recorded	0	0
Glady	Response to Intervention	Calm	0	0	Follow-up Mood	Euthymic	0	0
		Cooperative	0	0		Angry	20	1
		Paradoxical	33	1		Anxious	20	1
		Uncooperative	33	1		Irritable	40	2
		Hostile	33	1		Depressed	0	0
		Other	0	0		Labile	20	1
		Not recorded	0	0		Afraid	0	0
						Euphoric	0	0
						Other	0	0
						Not recorded	0	0
Ken	Response to Intervention	Calm	47	28	Follow-up Mood	Euthymic	0	0
		Cooperative	51	30		Angry	4	3
		Paradoxical	2	1		Anxious	29	22
		Uncooperative	0	0		Irritable	32	25
		Hostile	0	0		Depressed	5	4
		Other	0	0		Labile	30	23
		Not recorded	0	0		Afraid	0	0
						Euphoric	0	0
						Other	0	0
						Not recorded	0	0
Leonard	Response to Intervention	Calm	0	0	Follow-up Mood	Euthymic	0	0
		Cooperative	0	0		Angry	50	1
		Paradoxical	0	0		Anxious	0	0
		Uncooperative	100	1		Irritable	50	0
		Hostile	0	0		Depressed	0	0
		Other	0	0		Labile	0	0
		Not recorded	0	0		Afraid	0	0
						Euphoric	0	0
						Other	0	0
						Not recorded	0	0
Marcus	Response to Intervention	Calm	40	14	Follow-up Mood	Euthymic	0	0
		Cooperative	40	14		Angry	19	11
		Paradoxical	0	0		Anxious	15	9
		Uncooperative	6	2		Irritable	36	21
		Hostile	14	5		Depressed	5	3
		Other	0	0		Labile	24	14
		Not recorded	0	0		Afraid	0	0
						Euphoric	0	0
						Other	0	0
						Not recorded	2	1
Marshall	Response to Intervention	Calm	47	24	Follow-up Mood	Euthymic	0	0
		Cooperative	37	19		Angry	0	0
		Paradoxical	0	0		Anxious	17	8
		Uncooperative	12	6		Irritable	33	15
		Hostile	4	2		Depressed	0	0
		Other	0	0		Labile	50	23
		Not recorded	0	0		Afraid	0	0
						Euphoric	0	0
						Other	0	0
						Not recorded	0	0
Michael	Response to Intervention	Calm	100	2	Follow-up Mood	Euthymic	0	0
		Cooperative	0	0		Angry	0	0
		Paradoxical	0	0		Anxious	50	1
		Uncooperative	0	0		Irritable	0	0
		Hostile	0	0		Depressed	50	1
		Other	0	0		Labile	0	0
		Not recorded	0	0		Afraid	0	0
						Euphoric	0	0
						Other	0	0
						Not recorded	0	0
Tim	Response to Intervention	Calm	50	1	Follow-up Mood	Euthymic	0	0
		Cooperative	50	1		Angry	0	0
		Paradoxical	0	0		Anxious	33	1
		Uncooperative	0	0		Irritable	33	1
		Hostile	0	0		Depressed	0	0
		Not recorded	0	0		Labile	33	1
						Afraid	0	0
						Euphoric	0	0
						Other	0	0
						Not recorded	0	0

*Note.* Part. = participant; % Base = percentage base; Freq. = frequency.

##### Follow-Up Mood

The moods reported across participants varied (see Table 4). Irritability was observed 33% of the time for Bobby, while Glady displayed irritability 40% of the time. Ben and Clark exhibited anxious moods 27% and 30% of the time, respectively. Labile moods were most common for Gerald (54%) and Marshall (50%).

##### Challenging Behavior Severity Median at Intake

Ben and Marcus had the highest severity median scores of 4.00. This was followed by Bobby and Glady at 3.50. Clark and Gerald each had a median score of 3.00, while Ken and Marshall scored 2.50 and 2.00, respectively. Freddy and Tim received median severity scores of 1.50. Leonard and Michael received the lowest median scores at 1.00.

Table 5 outlines the results for restraint characteristics for continuous variables.

**Table 5.** Restraint Characteristics Summary for Continuous Variables.

Participants	Total frequency of restraint notes	Total frequency of restraints	Total frequency of restraint reapplication	Overall restraint rate (restraints/100 hr	Latency to first restraint (hours)
Ben	104	144	40	39.70	1.03
Bobby	4	4	0	0.60	58.80
Clark	14	14	0	1.80	5.30
Freddy	23	31	8	5.00	44.97
Gerald	68	92	24	37.60	11.03
Glady	2	3	1	2.20	63.35
Ken	31	34	3	10.70	130.98
Leonard	1	1	0	0.30	344.58
Marcus	24	26	2	4.30	10.23
Marshall	26	35	9	6.50	24.37
Michael	2	2	0	0.10	183.20
Tim	1	2	1	0.60	175.58

*Note.* This table shows the exact values for restraint characteristics. Overall restraint rate depicts the frequency of restraint applications per 100 hr of service.

##### Total Frequency of Restraint Notes

Overall, Ben had the highest number of restraint notes on record, totaling 104. In contrast, Leonard and Tim had only one restraint note each. The median frequency of restraint *notes* across the participants was 18.50.

##### Total Frequency of Restraints

Ben experienced the highest total number of restraints, at 144. This was followed by Gerald with 92 instances of restraints. Michael and Tim each experienced two restraints, while Leonard experienced only one. The median frequency of restraints was 20.

##### Total Frequency of Restraint Reapplication

Ben experienced restraint *re*application most often (i.e., re-initiation of a restraint shortly after release), with 40 instances. Gerald required 24 instances of reapplication, while Marshall experienced reapplication nine times. The remaining participants required reapplications fewer than nine times each. Michael, Ken, Clark, and Bobby did not require reapplications.

##### Overall Restraint Rate

The average restraint rate across participants was 9.11 per 100 service hours (range, 0.10–39.70 per 100 service hours).

##### Inter-Response Interval Time of Restraint Applications

Regarding this variable, Ben’s average inter-response interval duration was 2.63 hr (range, 0.08–63.45 hr). Bobby’s average inter-response interval was 162.91 hr (range, 0.47–581.88 hr). Clark’s average inter-response interval was 50.54 hr (range, 0.32–233.13 hr). Freddy’s average was 18.60 hr (range, 54.38–138.85 hr). Gerald’s average inter-response interval was 2.45 hr (range, 0.03–24 hr). Glady’s average was also 36.48 hr (range, 45.90–63.58 hr). Ken’s average was 5.59 hr (range, 8.83–130.98 hr). For Leonard, only one inter-response interval duration was recorded (14.82 s); therefore, an average duration could not be computed. Next, Marcus’s average inter-response time was 22.49 hr (range, 36.95–102.23 hr). Marshall’s average inter-response time was 12.71 hr (range, 0.57–97.88 hr). Michael’s average inter-response time to restraint was 685.56 hr (range, 19.10–1,352.03 hr). Finally, Tim’s average inter-response interval was also 88.21 hr (range, 0.10–176.32 hr).

##### Latency

The shortest latency to first restraint application was experienced by Ben at 1.03 hr, followed by Clark at 5.30 hr. The longest latency was experienced by Leonard at 344.58 hr, followed by Michael at 183.20 hr, Tim at 175.58 hr, and Ken at 130 hr. The average latency to first restraint application was 87.78 hr across participants.

### Regression Results and Corresponding Model Assessments

The dependent variable, latency to first restraint application, was assessed to determine whether it met parametric statistical assumptions (e.g., homogeneity, normality, linearity; Field, 2024). A Q-Q plot showed a notable deviation from normality, though skewness and kurtosis indicated only modest violations, with a large standard deviation (*SD* = 103.64). Given the small sample size (*N* = 12), reliance on the central limit theorem is not advisable (Field, 2024). A scatter plot suggested possible linearity issues, thus a log10 transformation was applied to address the assumption violations described above (see Friedel et al., 2022; West, 2022).

The regression analysis outcomes indicated that median challenging behavior severity score may significantly predict log10 transformed latency to first restraint (β = −.479, *p* = .017, 95% CI [−0.74, −0.21], *f*² = 0.90). That is, the relationship between the median challenging behavior severity score and latency to the first restraint application may be multiplicative in nature. Applied to the current case, where β = −.479, a one-unit increase in challenging behavior severity score (e.g., a score of 2 to 3) may coincide with a 67% decrease in latency to restraint application (calculated as 1–10^−0.479^ = 0.67). It follows that a two-unit increase in challenging behavior severity score (e.g., a score of 1 to 3) may coincide with an 89% decrease in latency to first restraint application (calculated as 1–10^−0.479 × 2^ = 0.89). Thus, these results suggest that for each unit increase in the severity score, the latency to restraint application decreases proportionally. Simply put, higher median challenging behavior severity scores may be associated with a decrease in latency to restraint application.

## Discussion

The current project has yielded several noteworthy outcomes and patterns that may invite future research. However, prior to describing and discussing these outcomes we felt it prudent to preface this section with the following caveat: our small sample size requires cautious interpretation of the results.

First, higher median challenging behavior severity scores may have been significantly associated with shorter latency to first restraint application. This finding may underscore the utility of incorporating structured severity metrics at intake (e.g., DBSS). For instance, clinicians may consider using structured approaches to identify clients with high-risk profiles because doing so could help improve their precision regarding decisions around proactively allocating additional resources, such as increased staff-to-client ratios or enhanced preventive programming. Relatedly, intake challenging behavior severity data may serve as clinical justification for requesting additional staffing or clinical supports to ensure safe and ethical implementation of emergency procedures. This outcome could also suggest it may be important for clinicians to track latency as part of ongoing client progress monitoring. Given these promising outcomes, next research steps may include examining challenging behavior severity scores at intake across those who experience restraint application and those who do not (i.e., control group) to determine whether the tool’s predictive capacity demonstrated in the current study persists (see Knottnerus & Buntinx, 2011 for an introduction to theory and methods of diagnostic research). Along these lines, future research may endeavor to collaborate with other agencies that use emergency restraints, and, through these joint initiatives, encourage agencies to adopt the DBSS as an intake tool. These efforts could support replication and extension (depending on the available sample size) of the current analysis. Finally, extending the current work could permit investigating other potential independent variables (e.g., participant size, number of psychotropic medications, response rate) resulting in a fulsome model of this phenomenon, including the examination of interaction effects where applicable.

Another interesting pattern was that higher restraint rates coincided with shorter service durations and greater staff involvement. As service duration increased, the total number of restraint notes decreased. This may indirectly speak to treatment gains. Specifically, participants’ challenging behavior episodes no longer necessitated restraint application, potentially reflecting positive programming effects. This outcome could be used as evidence in support of our decision to use latency to first restraint application as the dependent variable for the regression analysis. Future research may consider foregoing restraint rate as the dependent variable to minimize confounds associated with the passage of time (e.g., treatment gains). Potential clinical implications of this outcome could be that senior clinical team members charged with program evaluation (i.e., finding service delivery efficiencies) may consider adding latency to first restraint application as a data point to review across service recipients, as it may lend further insights over and above that of response rate.

Third, inter-response intervals patterns observed in the current project may suggest there could be a relationship between average inter-response interval and service duration. Specifically, as service duration increased, the average inter-response intervals increased. Again, these outcomes could be interpreted as a reflection of treatment gains. This finding suggests that there may be added value in extending the service provision duration for future cases with similar profiles. Given that applied behavior analytic treatment dosage research is relatively underdeveloped for individuals with severe challenging behavior who require restraint application, this may be an important area for research extension (see Linstead et al., 2017 for more on this topic).

Fourth, most participants may have been considered *older* adolescents (*M* = 15.25 years). Thus, it is possible that age may differentially influence emergency restraint application. That said, Ben (14 years old) required restraint application most often, yet he was not the eldest participant. Gerald was 19 years old and required a total of 92 restraints, 24 of which were reapplications. By contrast, Bobby (17 years old) required only four restraints, and Michael (19 years old) needed two restraints and did not require any reapplications. Collectively, this idiosyncratic pattern could suggest age may represent only a modest predictor, which may or may not be moderated by other variables (e.g., participant size, challenging behavior severity). We strongly encourage future researchers to bolster their sample size so that they can examine potential interactions between age and other clinically relevant variables, such as challenging behavior function, service setting, challenging behavior severity, client size etc.

A fifth notable pattern was the high prevalence of polypharmacy across the sample (83.33%). Although the current sample was small, this pattern may align with existing literature on polypharmacy in persons with intellectual and developmental disabilities who exhibit challenging behavior. Specifically, polypharmacy rates range from 68.2% to 100% (Lonchampt et al., 2021; O’Brien et al., 2024). Follow-up research may include investigating whether polypharmacy patterns significantly differ across those who engage in challenging behavior and experience restraint applications versus those who engage in challenging behavior but do not experience restraint application.

One final pattern we felt may be important to describe was that APNs reported most participants (i.e., 83.33%) appeared calm and engaged in cooperative behavior shortly after restraint application (i.e., Response to Intervention). This pattern also appeared to persist across individual restraint events. At times, challenging behavior treatment literature may underreport potential adverse side effects (Cramer et al., 2024; Heyvaert et al., 2015). Our outcomes may highlight the importance of recording and reporting potential adverse side effects that may coincide with behavior analytic interventions in general (whether reinforcement-based or otherwise). Improved side effect reporting may facilitate an enhanced understanding of the overall intervention impact (Bottema-Beutel et al., 2021). It is important to note that we are interpreting this outcome in the context of the specific setting, which was an outpatient clinic staffed by doctoral-level BCBAs, registered behavior technicians, and licensed medical professionals. All staff were extensively trained in physical management procedures and used a systematic, individualized approach to therapy. Thus, such outcomes may not generalize to other settings with different staffing or procedural rigor. Nonetheless, when applied carefully by skilled professionals with appropriate clinical oversight, restraint application in this context may have served a protective role, preventing further harm. Given how mood data were recorded (and corresponding limitations associated with indirect measure approaches), future research may leverage wearable physiological monitoring devices to assess affect and physiological changes before, during and after *clinically indicated* restraint application. Such data could improve understanding of the broader restraint impact. Regardless, our outcomes underscore the importance of a balanced, individualized approach to restraint application, particularly in high-risk situations. Taken together, the restraint application affect outcomes, alongside other patterns described above, may serve to reiterate the importance of investing in robust staff training as well as ongoing qualified clinical oversight paired with a structured approach to identifying challenging behavior severity early on.

### Study Limitations

As with any research project, it is prudent to describe study limitations. First, our small sample size limited statistical power and we were such that we were only able to include one potential independent variable. Restraints were also used infrequently across participants in general, making the sample small compared to the broader clinical population. That said, the resultant model did coincide with narrower confidence intervals that did not cross zero and a large effect size, suggesting the model may be relatively precise and clinically significant. Future research on the topic should endeavor to secure a larger sample size to examine other relevant independent variables that may be associated with latency to restraint application. For instance, through our descriptive analysis, we found that challenging behavior function patterns might suggest that certain functions (e.g., tangible and multiply controlled challenging behavior) were more common among participants who required more restraints. This pattern may tentatively suggest that socially mediated functions involving access to preferred stimuli, may be associated with an elevated risk of restraint in the current sample. It follows that, we recommend future researchers bolster sample size so that additional predictors, specifically challenging behavior function (e.g., escape, attention, tangible) may be included in the statistical model. This may help to uncover whether certain consequences may be associated with restraint application. And, as such behavior function may represent a promising and underexplored variable in restraint research that may hold considerable value for informing both policy and practice.

Another study limitation may be related to the availability of data. That is, the analyses were limited to the information provided by the partnering agency. Admittedly, this could mean there may have been other worthwhile variables that could not be examined. However, we argue it is unlikely given how exceptionally comprehensive the restraint notes were. Additionally, due to resource constraints, only one APN collected data, and regulatory requirements mandated that only an APN or physician could complete restraint notes. As a result, IOA outcomes for restraint documentation were not available. Future studies examining restraint use systematically should consider involving a second professional to assess IOA during restraint events. In closing, the current study may have yielded some encouraging results and may offer viable next steps for future researchers interested in furthering work on this understudied area.

## Conclusions

The overarching purpose of the current paper was to demonstrate how behavior analytic researchers may leverage analyses to develop a predictive technology that may help prevent the need for restraint application. While a comprehensive discussion of restraints and associated risks is beyond the scope of the current paper, it is important to reiterate that restraint application carries inherent risks for implementers and service users (including lethal outcomes). It follows that decisions and discussion around restraint application should include all stakeholders to first determine whether the approach is necessary, appropriate and can be safely administered. These conversations should be guided by ethical and legal parameters (e.g., BACB, 2020; Vollmer et al., 2011), and if treatment teams decide to proceed with the application—restraints should be faded as quickly and safely as possible.

## Supplemental Material

sj-docx-1-bmo-10.1177_01454455261434863 – Supplemental material for Factors Associated With Restraint Application in Children and Adolescents With Intellectual and Developmental Disabilities Displaying Severe Challenging Behavior Supplemental material, sj-docx-1-bmo-10.1177_01454455261434863 for Factors Associated With Restraint Application in Children and Adolescents With Intellectual and Developmental Disabilities Displaying Severe Challenging Behavior by Asude Sumeyye Ayvaci, Alison Dorothea Cox and Daniel Mitteer in Behavior Modification
