# Supplementary material for: Factors Associated With Restraint Application in Children and Adolescents With Intellectual and Developmental Disabilities Displaying Severe Challenging Behavior
Source: Behav Modif. 2026 Apr 15;50(5):385–422. doi: 10.1177/01454455261434863 (PMC13424904; doi:10.1177/01454455261434863)
Supplement: sj-docx-1-bmo-10.1177_01454455261434863 – Supplemental material for Factors Associated With Restraint Application in Children and Adolescents With Intellectual and Developmental Disabilities Displaying Severe Challenging Behavior [file sj-docx-1-bmo-10.1177_01454455261434863.docx]

**Supplementary Materials**

**Table 1**

*Summary of Challenging Behavior Function by Topography Across Participants*

| Participant | Topography | Corresponding Behavior Function |
| --- | --- | --- |
| Ben | Aggression | Multiply Controlled (i.e., escape and tangible) |
|  | Disruption | Automatic |
|  | SIB | Automatic |
| Bobby | Aggression | Multiply Controlled (i.e., escape, attention and tangible) |
|  | Disruption | Multiply Controlled (i.e., escape, attention and tangible) |
| Clark | Aggression | Multiply Controlled (i.e., attention and tangible) |
|  | Property Destruction | Multiply Controlled (i.e., attention and tangible) |
|  | SIB | Multiply Controlled (i.e., attention and tangible) |
| Freddy | Aggression | Multiply Controlled (i.e., escape and tangible) |
|  | Property Destruction | Multiply Controlled (i.e., escape and tangible) |
| Gerald | Aggression | Multiply Controlled (i.e., escape, tangible, attention) |
|  | Property Destruction | Multiply Controlled (i.e., escape, tangible, attention) |
| Glady | NA | NA |
| Ken | Aggression | Multiply Controlled (i.e., Tangible, escape, social avoidance, mand denial) |
|  | Property Destruction | Multiply Controlled (i.e., Tangible, escape, social avoidance, mand denial) |
|  | SIB | Multiply Controlled (i.e., Tangible, escape, social avoidance, mand denial) |
| Leonard | Aggression | Multiply Controlled (i.e., escape, attention, tangible) |
|  | Property Destruction | Multiply Controlled (i.e., escape, attention, tangible) |
| Marcus | Aggression | Tangible |
|  | Property Destruction | Tangible |
| Marshall | Aggression | Multiply Controlled (i.e., attention, tangible [edibles]) |
|  | Property Destruction | Multiply Controlled (i.e., attention, tangible [edibles]) |
|  | SIB | Multiply Controlled (i.e., attention, tangible [edibles]) |
| Michael | Aggression | Tangible (i.e., edible) |
| Tim | Aggression | Undifferentiated (i.e., target behavior not observed) |
|  | SIB | Undifferentiated (i.e., target behavior not observed) |

*Note.* The table above outlines the behaviour function per challenging behavior topography across participants reported by the partnering agency, which was obtained via the implementation of a functional analysis. N/A = not applicable (i.e., not recorded). SIB = self-injurious behavior.

**Table 2**

*Number of Support Staff Involved Across All Restraint Notes*

| Participant | Restraint Note Total | Category | Frequency | Percentage |
| --- | --- | --- | --- | --- |
|  |  | Number of Staff Involved |  |  |
| Ben | 104 | 1–2 | 19 | 18.27% |
|  |  | >2 | 85 | 81.73% |
| Bobby | 4 | 1–2 | 1 | 25% |
|  |  | >2 | 3 | 75% |
| Clark | 14 | 1–2 | 3 | 21.43% |
|  |  | >2 | 11 | 78.57% |
| Freddy | 23 | 1–2 | 1 | 4.35% |
|  |  | >2 | 22 | 95% |
| Gerald | 68 | 1–2 | 0 | 0% |
|  |  | >2 | 68 | 100% |
| Glady | 2 | 1–2 | 1 | 50% |
|  |  | >2 | 1 | 50% |
| Ken | 31 | 1–2 | 4 | 12.90% |
|  |  | >2 | 27 | 87.10% |
| Leonard | 1 | 1–2 | 1 | 100% |
|  |  | >2 | 0 | 0% |
| Marcus | 24 | 1–2 | 1 | 4.17% |
|  |  | >2 | 23 | 95.83% |
| Marshall | 26 | 1–2 | 5 | 19.23% |
|  |  | >2 | 21 | 80.77% |
| Michael | 2 | 1–2 | 1 | 50% |
|  |  | >2 | 1 | 50% |
| Tim | 1 | 1–2 | 0 | 0% |
|  |  | >2 | 1 | 100% |

*Note.* This table summarizes the number of support staff involved across all restraint notes per participant where one or more emergency restraints were applied at the onset of severe challenging behavior.
